# Release behavior and toxicity profiles towards A549 cell lines of ciprofloxacin from its layered zinc hydroxide intercalation compound

**DOI:** 10.1186/1752-153X-7-119

**Published:** 2013-07-12

**Authors:** Ahmad Faiz Abdul Latip, Mohd Zobir Hussein, Johnson Stanslas, Charng Choon Wong, Rohana Adnan

**Affiliations:** 1 Materials Synthesis and Characterization Laboratory (MSCL), Institute of Advanced Technology (ITMA), Universiti Putra Malaysia UPM, 43400 Serdang, Selangor, Malaysia; 2 Pharmacotherapeutics Unit, Department of Medicine, Faculty of Medicine and Health Sciences, Universiti Putra Malaysia UPM, 43400 Serdang, Selangor, Malaysia; 3 School of Chemical Sciences, Universiti Sains Malaysia, 11800 Pulau, Pinang, Malaysia

**Keywords:** Drug delivery, Layered zinc hydroxide nitrate, Ciprofloxacin, Anion exchange, Sustained release, Release mechanisms, Cytotoxicity

## Abstract

**Background:**

Layered hydroxides salts (LHS), a layered inorganic compound is gaining attention in a wide range of applications, particularly due to its unique anion exchange properties. In this work, layered zinc hydroxide nitrate (LZH), a family member of LHS was intercalated with anionic ciprofloxacin (CFX), a broad spectrum antibiotic via ion exchange in a mixture solution of water:ethanol.

**Results:**

Powder x-ray diffraction (XRD), Fourier transform infrared (FTIR) and thermogravimetric analysis (TGA) confirmed the drug anions were successfully intercalated in the interlayer space of LZH. Specific surface area of the obtained compound was increased compared to that of the host due to the different pore textures between the two materials. CFX anions were slowly released over 80 hours in phosphate-buffered saline (PBS) solution due to strong interactions that occurred between the intercalated anions and the host lattices. The intercalation compound demonstrated enhanced antiproliferative effects towards A549 cancer cells compared to the toxicity of CFX alone.

**Conclusions:**

Strong host-guest interactions between the LZH lattice and the CFX anion give rise to a new intercalation compound that demonstrates sustained release mode and enhanced toxicity effects towards A549 cell lines. These findings should serve as foundations towards further developments of the brucite-like host material in drug delivery systems.

## Background

The application of nanotechnology to drug delivery is nowadays a growing research field. A wide variety of nano-sized drug carriers has found niche in the field, owing to their unique structures which give rise to new generations of therapeutic agents and medical devices [1]. The main advantages of the nano-based drug delivery over the traditional ones are manifold: enhanced biodistribution and pharmacokinetics of drug [2], improved delivery of poorly water-soluble drugs [3], lowered systemic toxicity of drug while being concentrated on the target organ [4] and ability to optimize drug release rate towards achieving better patient compliance [5].

Layered hydroxides salts (LHS) is a layered inorganic compound which shares structural resemblance to anionic clay, layered double hydroxides (LDH). The structure of LDH is derived from that of brucite, [Mg(OH)_2_] and may be represented by the formula [M^2+^_1-x_M^3+^_x_ (OH)_2_](A^n-^)_x/n_.mH_2_O; where M^2+^ and M^3+^ are divalent and trivalent cations of the lattice, respectively, x is equal to the ratio [M^3+^/( M^2+^ + M^3+^)] and A^n-^ is an anion [6]. In relation to LDH, its LHS sibling may undergo structural modifications based on different type of metal cation that is present in the compound lattice. It has been reported that nitrate group precursor are directly involved in the formation of LHS of Cu_2_(OH)_3_NO_3_, La(OH)_2_NO_3._H_2_O and Mg_2_(OH)_3_NO_3_ via coordination with the lattice cation through one oxygen atom of the nitrate ion [7,8].

In Zn_5_(OH)_8_(NO_3_)_2_.2H_2_O (denoted as LZH), the brucite-like lattice is modified wherein one-fourth of octahedrally coordinated Zn^2+^ cations are absent, thus creating empty octahedral sites. On either side of the empty octahedra are found tetrahedrally coordinated Zn^2+^ cations with the hydroxyl ions and water molecules. In this compound, the nitrate ion is not coordinated with the Zn^2+^ cations and located in the interlayer space of LZH [9].

LHS is currently gaining attraction due to its simple method of synthesis [10], as a precursor for a wide band gap ZnO [11], for the synthesis of layered double hydroxide salts [12] and anion exchange properties [13]. A wide variety of guest molecules has been intercalated into the interlayer region of LHS, mainly via ion exchange process, ranging from anionic dyes [14], porphyrin sensitizers [15] and an anti corrosive compound [16]. In particular, LHS has demonstrated the ability to extend the release period of bioactive molecules [17] and drug molecules [18], prompting more investigations towards potential applications of LHS in drug delivery systems.

Ciprofloxacin (CFX) is a wide spectrum antibiotic that belongs to the quinolone family [19]. The antimicrobial activities of CFX are mainly achieved through the chlorine-substituted N–1 cyclopropyl group which enhances cell penetration and improves activity against DNA gyrase and topoisomerase IV enzymes [20]. Although CFX is known as a safe drug, there are cases of side effects associated with CFX such as anaphylaxis and pulmonary edema [21,22]. CFX suffers from moderate oral bioavailability [23], as it chelates with calcium-, magnesium- and aluminium-containing salts upon concomitant administration [24]. In drug delivery systems, CFX has been used with various drug carriers such as polymeric nanoparticles [25-27], cyclodextrin [28], chitosan [29], montmorillonite [30] and calcium apatite [31].

In this paper, we prepared an inorganic drug carrier based on LZH host material intercalated with a model drug, CFX. Considering LZH possesses higher layer charge density compared to that of LDH counterpart [32], we are prompted to examine the release behavior of the intercalated CFX anions from LZH in phosphate-buffered saline solution, after which the corresponding release mechanisms was further established. In addition, the toxicity profile of the intercalation compound was evaluated against adenocarcinomic human alveolar basal epithelial cancer cell line to demonstrate synergistic effects between drug–host interactions towards cells growth inhibition [33].

## Materials and methods

### Materials

Ciprofloxacin, C_17_H_18_FN_3_O_3_ (1–cyclopropyl–6–fluoro–4–oxo–7–piperazin–1–yl–quinoline–3–carboxylic acid, molecular weight 331.34 g/mol) was purchased from Sigma Aldrich Co. Ltd. and was used as received. All solutions were prepared using deionized water.

### Synthesis of LZH

Layered zinc hydroxide nitrate (LZH) was synthesized according to the modified version of previous report [32]. An aqueous solution of 0.4 mol/L Zn(NO_3_)_2_.6H_2_O was prepared in 100 ml volumetric flask. To this solution, 0.8 mol/L NaOH solution was added dropwise, under vigorous magnetic stirring, until pH of the mixture reached pH 7.0. The resulting precipitates were aged at 70°C for 18 h, washed thoroughly with deionized water and dried in an oven at 60°C.

### Synthesis of Z–CFX

The intercalation compound, Z–CFX was obtained via anion exchange between nitrate ion of precursor LZH and anionic ciprofloxacin (CFX) in a mixture solution of water:organic solvent. Approximately 0.2 g of finely ground LZH was dispersed in 25 ml of water:ethanol mixture solution containing 0.9 g of CFX under vigorous stirring. The pH of the exchange medium was adjusted by slow titration of 1.0 mol/L NaOH until pH 8.0 was achieved. The mixture was left under stirring for 24 h. The resulting product was collected by washing the precipitates thoroughly with deionised water and ethanol and was dried at 60°C for 24 h.

### In vitro release

The release of CFX from the intercalation compound was conducted in phosphate-buffered saline solution (PBS) pH 7.4 wherein 0.6 mg of Z–CFX were immersed in the PBS solution and the accumulated release of CFX was measured at λ_max_ = 276.3 nm using a Perkin Elmer UV–Vis Spectrophotometer Lambda 35.

### Cell culture

Human lung alveolar carcinoma epithelial (A549) cells were cultured in RPMI 1640 medium under a humidified atmosphere (5% CO_2_ plus 95% air) at 37°C. The medium was supplemented with 10% heat-inactivated fetal bovine serum, 2 mM of L–glutamine, 100 units/ml of penicillin and 100 μg/ml of streptomycin.

### MTT assay

MTT (3–(4,5–dimethylthiazole–2–yl)–2,5–diphenyltetrazolium bromide) cell viability assay was used to investigate the toxic effect of Z–CFX, ZLH and CFX. Cells (2 × 10^3^ cells/100 μl) were seeded onto 96-well plates and incubated overnight at 37°C under a 5% CO_2_ atmosphere. After cells had stabilized, fresh medium containing either Z–CFX, ZLH or CFX at different concentrations (0.5, 5.0, 50.0 and 500.0 μg/mL) was added and incubation continued for 72 h. After the incubation, 10 μL of MTT solution was added to each well and incubated further for 4 h, the reaction was terminated by adding 100 mL of 10% SDS in 0.01 mol/L HCl solution. The absorbance was measured on a microplate reader at wavelength 570 nm.

### Characterizations

Powder x-ray diffraction (PXRD) patterns were recorded on an ITAL APD 2000 powder diffractometer using CuK_α_ radiation (λ = 1.5418 Å) at 40 kV and 30 mA. The data was collected from 2–60° at a dwell time of 2° per minute. Fourier transform infrared (FTIR) spectra were recorded in the range 400–4000 cm^-1^ at a 4 cm^-1^ resolution on a Perkin–Elmer 1752X (Boston, MA) spectrophotometer using the potassium bromide (KBr) pellet technique; approximately 1% sample was mixed in 100 mg of spectroscopic grade KBr and the pellet was pressed at 10 tons. The atomic weight percent of carbon, hydrogen and nitrogen was determined using CHNS–932 (LECO Instruments St Joseph, MI). The zinc ion composition was determined using a Perkin–Elmer inductively coupled plasma-atomic emission spectrometry (ICP–AES) model Optima 2000DV under standard conditions. Thermogravimetric and differential thermogravimetric analyses (TGA/DTG) were carried out in 150 μL alumina crucibles using a Metter–Toledo instrument model TGA851e (Greifensee, Switzerland) at a heating rate of 10° per minute in the range of 25–900°C with the sample amount being 5–10 mg in nitrogen atmosphere. The surface morphology of the samples was observed by a field emission scanning electron microscope (FESEM), FEI Nova Nanosem 230 with an acceleration voltage of 25 kV. Prior to analysis, the samples were mounted on aluminum stub over double-coated carbon film. Textural characterisations were carried out using a nitrogen gas adsorption–desorption technique at 77 K using a Micromeritics ASAP2000. The sample was degassed in an evacuated heated chamber at 100°C overnight. Pore size distributions were calculated using the Barrett-Joyner-Halenda (BJH) model on the desorption branch.

## Results and discussions

### X-ray diffraction analysis

**Figure 1:**
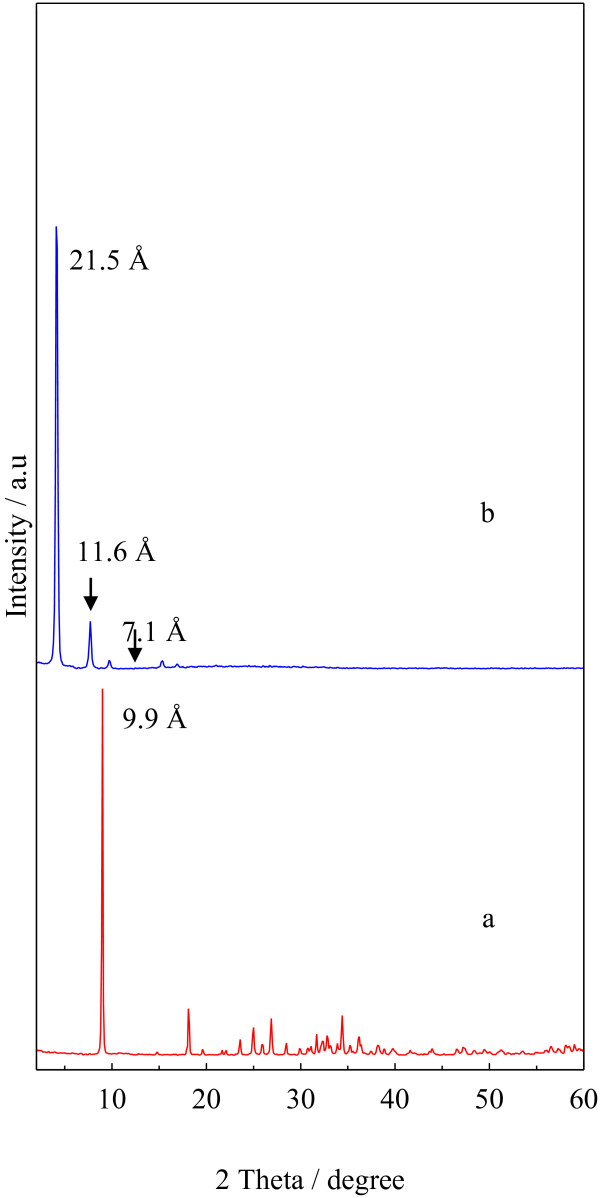
**XRD patterns of (a) LZH and (b) Z-CFX. (a)** host material, LZH. **(b)** intercalation compound, Z-CFX.

**Figure 2:**
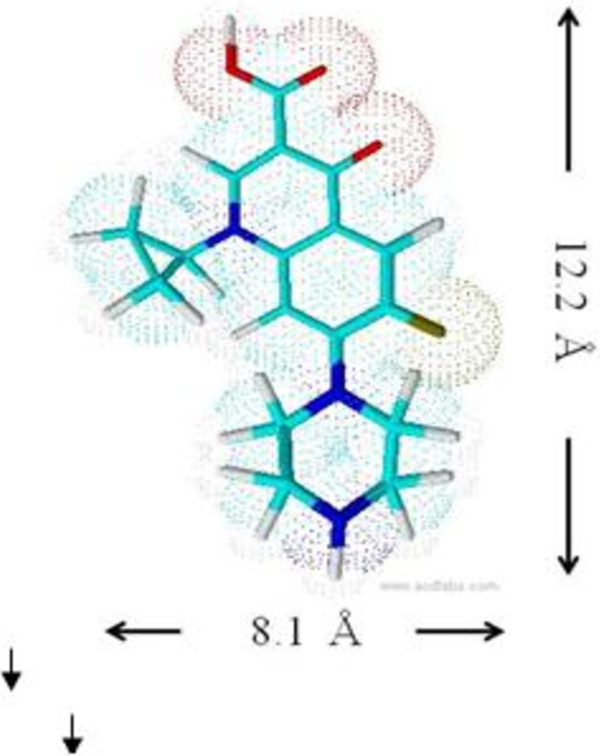
Molecular dimensions (including van der Waals radii) of CFX molecule.

**Figure 3:**
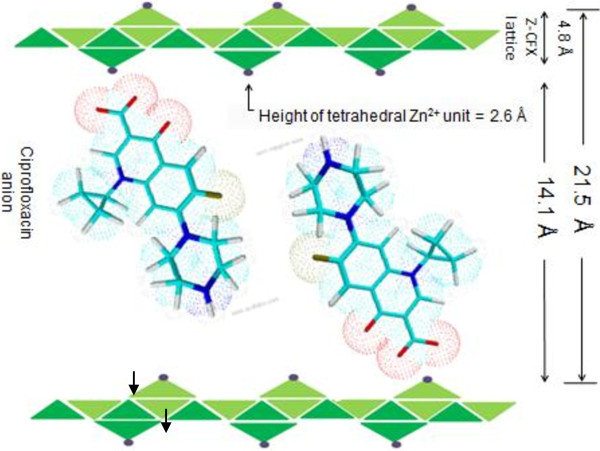
Schematic arrangement of CFX anions in the interlayer space of Z-CFX.

Figure 1 shows XRD patterns of LZH host material and Z–CFX intercalation compound. The diffraction patterns are characteristic of lamellar solid materials as indicated by sharp, intense basal reflections at low 2θ values and weaker non-basal reflections at higher angles [10]. Figure 1a displays the XRD pattern typical of LZH intercalated with nitrate ions [34]. The basal reflections of LZH are shifted to lower 2θ values as anion exchange is completed, indicating the formation of ciprofloxacin–LZH intercalation compound. Accordingly, the basal spacing of LZH expands from 9.9 Å to 21.5 Å in Z–CFX (Figure 1b). Therefore, the interlayer height of Z–CFX is estimated to be about 14.1 Å; obtained by subtracting the layer thickness plus the height of Zn^2+^ moiety of the lattice from the basal spacing; i.e. 14.1 Å = 21.5 – (4.8 + 2.6) Å. The obtained value is larger than the longitudinal length of CFX molecule (Figure 2). Therefore, we propose CFX anions were arranged as intertwined bilayers in the interlayer space [35], wherein the carboxylate groups of CFX were bonded through an oxygen atom to Zn^2+^ units of the lattice [7]. The proposed arrangement of the intercalated CFX anions in the interlayer space of LZH is illustrated in Figure 3.

### Fourier transform infrared spectroscopy

**Figure 4:**
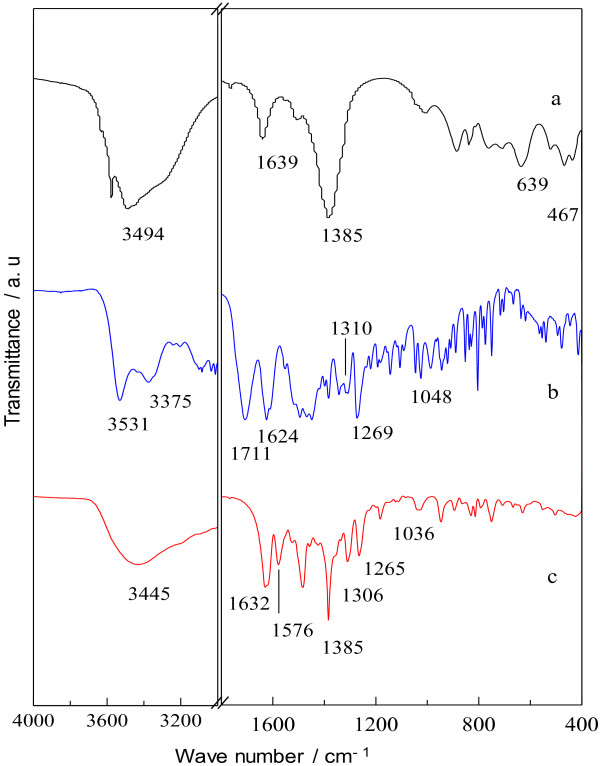
**FTIR spectra of (a) LZH, (b) CFX and (c) Z-CFX. (a)** host material, LZH. **(b)** free drug molecule, CFX. **(c)** intercalation compound, Z-CFX.

Figure 4 shows FTIR spectra of LZH host material, CFX molecule and Z–CFX intercalation compound. Only the main absorption bands are listed for the sake of clarity. In all spectra, broad absorption bands are observed in the range of 3440–3540 cm^-1^ due to the stretching vibrations of hydroxyl group of the lattice and water molecules. Figure 4a shows a typical FTIR spectrum of the host material, LZH with nitrate being the counter anion. An absorption band at 1639 cm^-1^ is attributed to the bending mode of water molecules [36]. At low frequency, bands arising from the lattice vibrations of Zn–O and O–Zn–O are detected at 639 and 467 cm^-1^, respectively. The most intense absorption band in LZH is found at 1385 cm^-1^, which is characteristic of free interlayer nitrate group (symmetry D_3h_) [8].

For CFX (Figure 4b), an absorption band at 3375 cm^-1^ is attributed to the stretching vibrations of amine group. Intense bands at 1711 and 1624 cm^-1^ are characteristic of the stretching vibrations of carbonyl group of carboxylic acid and ketone, respectively. Bands centered at 1310, 1269 and 1048 cm^-1^ are assigned to the stretching modes of C–N, C–C–C of ketone and C–F, respectively.

The FTIR spectrum of Z–CFX features main characteristic absorption bands of CFX anions which indicate that the anions were successfully intercalated into the LZH interlayers. Figure 4c depicts the stretching bands of asymmetric and symmetric of carboxylate group of the CFX anions, observed at 1576 and 1385 cm^-1^, respectively. Generally, difference in wavenumber between the carboxylate stretching bands (∆υ = υ_asym_ – υ_sym_) gives information about the coordination environment of the functional group. Li et al. [36] mentioned that carboxylate group adopting unidentate coordination mode has a larger ∆υ value compared to that of bridging carboxylate; 200 and 150 cm^-1^, respectively. Since the ∆υ of COO^–^ of CFX anions is 191 cm^-1^, we would suggest that the intercalated CFX is coordinately bonded to Zn^2+^ units of the lattice via one oxygen atom of the functional group.

The spectrum of Z–CFX also displays the other characteristic bands of CFX that were shifted from their initial positions as a result of multiple chemical interactions; electrostatic interactions between CFX anions and LZH lattice [18], as well as hydrogen bonding effect between water molecules and CFX anions [37]. The assignment of the absorption bands in the FTIR spectra of LZH, CFX and Z–CFX is summarized in Table 1.

**Table 1:** Assignment of FTIR absorption bands of Z-CFX, CFX and LZH

**Assignment**	**cm**^**-1**^		
	**LZH**	**CFX**	**Z-CFX**
υ(OH) of lattice, υ(O–H) COOH, υ(N–H) NH	3670 – 3200	–	3670 – 3200
υ(COOH)	–	1711	–
δ(OH) of H_2_O	1639 w	–	–
υ(C = O) ketone	–	1624 s	1632 s
υ_asym_(COO^–^)	–	–	1579
υ(NO_3_^–^)	1385 s	–	1385 sup
υ_sym_(COO^–^)	–	–	1385 sup
υ(C–N)	–	1310 m	1306 m
υ(C–C–C) ketone	–	1269 m	1265 m
υ(C–F)	–	1048 m	1036 w

Abbreviations: *S *sharp, *M *medium, *W *weak, *Sup* superimposed.

Table 2 summarizes the elemental analysis data for Z–CFX and LZH obtained from the CHN analysis and the corresponding stoichiometric formula for both samples. Approximately 42.63% of CFX was intercalated into the LZH interlayers as determined from the carbon content. Note that there are a small percentage of nitrate in the intercalation compound which indicates the total anion exchange was not achieved. Nonetheless, the stretching band of the remaining nitrate at 1385 cm^-1^ is not observed since it may have been obscured by the symmetric band of carboxylate of CFX anions [38]. There is also a small percentage of carbonate anions in the precursor LZH and in the intercalation compound Z–CFX, a feature commonly observed in anionic clays due to strong affinity of the anions towards highly positively charged clay lattice [39,40]. The normal absorption band of carbonate ion within 1360–1380 cm^-1^ maybe overlapped with the stretching vibration of nitrate ion and the symmetric vibration of the carboxylate group of CFX [41].

**Table 2:** Elemental analysis data, chemical formula and textural properties of LZH and Z-CFX

**Sample proposed formula**	**Weight percentage (%)**					**BET surface area (m**^**2**^**/g)**	**Pore volume (cm**^**3**^**/g)**	**Average pore diameter (nm)**
	**C**	**H**	**N**	**H**_**2**_**O**	**Zn**			
LZH	0.38	1.57	3.20	11.4	70.67	14.20	0.14	38.79
**Zn**_**5**_**(OH)**_**8**_**(NO**_**3**_**)**_**1.02**_**(CO**_**3**_**)**_**0.07**_**.2.93H**_**2**_**O**								
Z-CFX (Molecular weight: 872.53 g/mol)	26.27	3.27	6.17	12.33	45.26	26.51	0.17	25.94
**Zn**_**5**_**(OH)**_**8**_**(C**_**17**_**H**_**18**_**FN**_**3**_**O**_**3**_**)**_**0.93**_**(NO**_**3**_**)**_**0.14**_**(CO**_**3**_**)**_**0.07**_ **· 4.92H**_**2**_**O**								

### Thermal analysis

**Figure 5:**
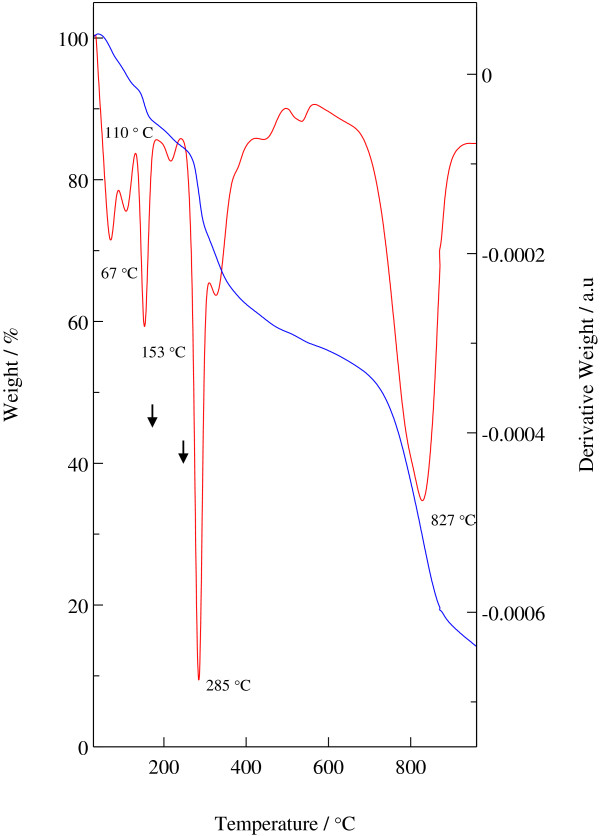
**TGA and DTG profiles of Z-CFX.** Blue line: TGA profile. Red line: DTG profile.

Figure 5 shows TGA and DTG profiles of Z–CFX. The thermal decomposition of Z–CFX follows the general route observed in LHS intercalated with organic anions [42]. The first step occurs from ambient up to 200°C with respective to the weight loss of 12.5% which is due to the removal of adsorbed and intercalated water. The compound further undergoes a 26% of weight loss in the region of 240–500°C which is attributed to the dehydroxylation of the hydroxide layers as well as partial decomposition of the intercalated CFX anions. The final step records a 35% weight loss with the major peak occurs around 827°C (temperature range 680–928°C) which is ascribed to the complete decomposition of amorphous mixture of salts generated during the initial drug decomposition [15].

### Textural analysis

**Figure 6:**
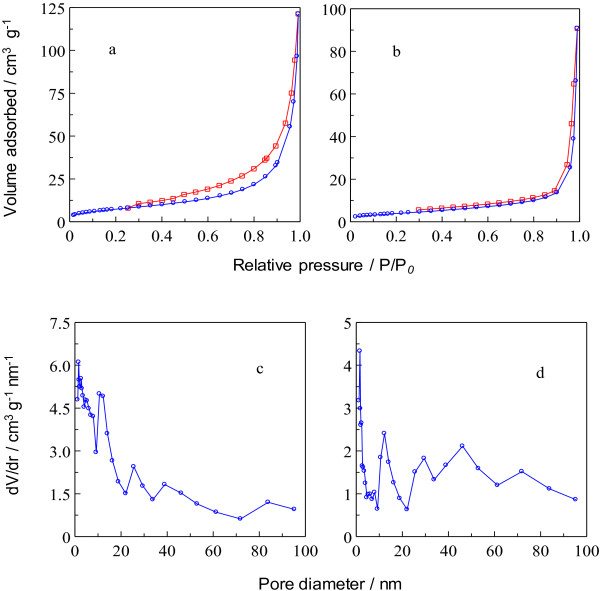
**Nitrogen adsorption–desorption isotherms of (a) Z-CFX and (b) LZH, and pore size distribution of (c) Z-CFX and (d) LZH. (a)** nitrogen adsorption–desorption isotherm of Z-CFX. **(b)** nitrogen adsorption–desorption isotherm of LZH. **(c)** pore size distribution of Z-CFX. **(d)** pore size distribution of LZH.

Figure 6a illustrates Type IV adsorption–desorption isotherm following the IUPAC classification which reveals the mesoporous nature of Z–CFX. The adsorption isotherm elucidates monolayer adsorptions commenced on the pore surface at low partial pressures followed by multilayer formation up to high partial pressures. There was no limiting adsorption of N_2_ gas observed at high partial pressures suggesting the presence of macropores [43]. This finding is in agreement with the wide distribution of pore size in Figure 6c, ranging from 1–95 nm, whereas the maximum size distribution being 2 nm. The hysteresis loop, indicative of capillary condensation in the mesopores occurred on the desorption isotherm down to partial pressure of around 0.25. The hysteresis loop which belongs to Type H3 hysteresis loop is characteristic of aggregates of plate-like particles [44].

Table 2 summarizes the specific surface area (SSA), the pore volume and the average pore diameter for LZH and Z–CFX as determined from the Brunaeur, Emmett and Teller (BET) method and the Barrett, Joyner and Halenda (BJH) method. It is worth mentioning that the CFX-intercalated LZH shows a larger surface area of 27 m^2^/g compared to that of the host with nitrate as the counter anion, LZH which is 14 m^2^/g. This finding is dissimilar from another group which observed the decreased in surface area value of LDH after being intercalated with organic anions [45]. Moreover, reports on the N_2_ adsorption–desorption of LZH intercalated with drug anions are rather scarce for comparison purposes with our aforesaid findings [46].

Recently, Hussein and co-workers reported that surface area of hippurate–LZH intercalation compound was decreased compared to that of the starting material, ZnO [47]. Hippuric acid was first dissolved in dimethyl sulfoxide before it was added to the ZnO suspension. On the contrary, in this work, CFX was dissolved in a mixture of water:ethanol solution to solubilize the drug prior to its intercalation into LZH since the drug CFX has poor solubility in aqueous solution. In a related finding, Malherbe et al. [48] showed that the surface area of hexacyanoferrate-intercalated LDH had increased when the intercalation compound was obtained via anion exchange in water-organic solvent mixtures. The group concluded that the inherent properties of organic solvents were responsible for the increased surface area of the obtained materials. We would attribute the increased in surface area of Z–CFX compared to LZH is due to different pore texture of the resulting material, which is very much depending on the method of synthesis.

### Morphology analysis

**Figure 7:**
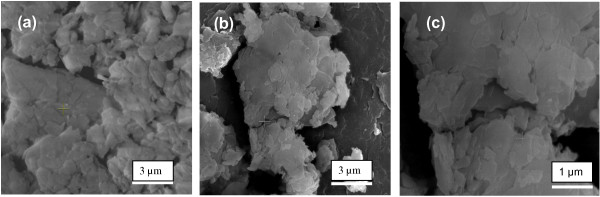
**SEM images of (a) LZH (b) Z-CFX (c) Z-CFX observed at high resolution. (a)** host material, LZH. **(b)** intercalation compound, Z-CFX.

Figure 7 depicts the FESEM micrographs of the hybrid material, Z–CFX and the host material, LZH. In Figure 7a, LZH exhibited aggregated plate-like particles which are stacked on top of each other. The intercalation of CFX molecules into the host, however, did not significantly change the morphology of Z–CFX (Figure 7b). Yang et al. [32] observed similar phenomenon in which no appreciable changes are observed in the plate-like morphology of the indole-3–acetic–intercalated zinc layered hydroxides and the parent layered zinc hydroxides.

### Release study

**Figure 8:**
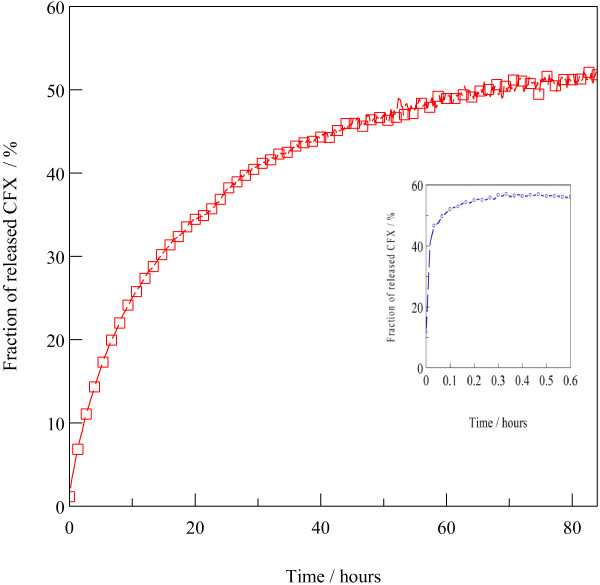
**Release profile of CFX from Z-CFX in PBS pH 7.4 and from physical mixture of LZH-CFX (inset).** Red line: release profile of CFX from Z-CFX. Blue line: release profile of physical mixture of LZH-CFX.

Release of drug anion from the hybrid material was done in the PBS solution pH 7.4 to evaluate its potentials as a drug carrier. Figure 8 shows the release profile of CFX which was achieved in a slow, sustained behaviour over 80 h that was in contrast with that of the physical mixture which reached an equillibrium within 20 min (inset Figure 8). The exceptionally slow, sustained release of Z–CFX was ascribed to the coordination bond between tetrahedral Zn^2+^ units with the intercalated anions as determined from the FTIR spectra (Figure 4). CFX anions were strongly held to the extent that their diffusion from the interlayer into the exchange medium solution was retarded, owing to the fact that total release of CFX was not achieved (CFX release at equillibrium was approximately 56%).

In order to gain insights into the mechanisms of CFX release from Z–CFX, we have applied four kinetic models commonly applicable in the kinetic study of drug-intercalated layered hydroxides hybrids. The models are:

1. First-order model which demonstrates the release system where dissolution rate depends on the amount of drug present in the intercalation compound and can be mathematically expressed as [49]:

$$\text{log}\left(C_{t}/C_{o}\right)=-K_{1}t$$

2. Parabolic diffusion model which describes the diffusion-controlled release of a drug from a medium and is generally written as [50]:

$$\left(1–C_{t}/C_{o}\right)/t=\mathrm{Kd}t^{–0.5}+a$$

3. The modified Freundlich model which explains experimental data on ion exchange and diffusion-controlled process following the equation [51]:

$$\left(C_{o}–C_{t}\right)/C_{o}=K{\mathrm{mt}}^{b}$$

4. The Bhaskar model which deals with the diffusion-controlled release of drug from particles and is summarised in the form [52]:

$$-\text{log}\left(1-C_{t}/C_{o}\right)=t^{0.65}$$

In equations 1 – 4, C_o_ and C_t_ are the amount of drugs in the LZH matrix at release time 0 and t, respectively, K is the rate constant, and a and b are the constants whose chemical significance is not clearly understood [51].

**Figure 9:**
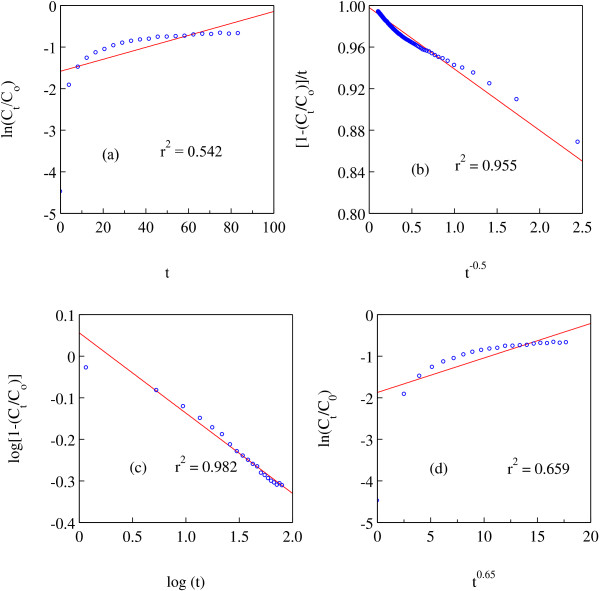
**Release data of CFX from Z-CFX into PBS solution pH 7.4 Fitted to four kinetic models. (a)** first order model, **(b)** parabolic diffusion model, **(c)** modified freundlich model, **(d)** bhaskar model.

The release data of CFX were fitted to the above models and the corresponding linear correlation coefficients (R^2^) were obtained and compared in Figure 9. The first-order model seems to be incompatible for describing the mechanisms due to low R^2^ value. The model yields poor linearity because it did not take into account the inherent complexity involved in the release process (R^2^ = 0.54). The Bhaskar model also gives a poor linearity (R^2^ = 0.66) as the model did not deal with the possibilities of both dissolution of the inorganic host and the anion exchange of the hybrid [52].

The fitting of release data is best achieved with the modified Freundlich model (R^2^ = 0.98), followed by parabolic diffusion (R^2^ = 0.96) which suggest that the release process is of diffusion-controlled. Note that high R^2^ value of the latter model is due to the “grouping” of the data towards low values on the *x*-axis, which is often observed upon applying this model for the kinetic analysis in layered hydroxides [18,53]. Generally, there are two governing mechanisms in the release system of layered double hydroxides (LDH); anion diffusion through particles and dissolution of the LDH particles [49]. The modified Freundlich model which concerns with the heterogeneous diffusion from flat surfaces via ion exchange would describe better the release process in Z–CFX; surface CFX anions diffused first into the PBS medium and underwent exchange with phosphate ions in the medium. The process was followed by diffusion from the interlayer anions. The latter process being designated as the rate limiting step [51]. We would attribute the sustained release of CFX anions due to the strong coordination bond which occurred between the anions and the Zn ions of the LZH lattice.

### Toxicity study

**Figure 10:**
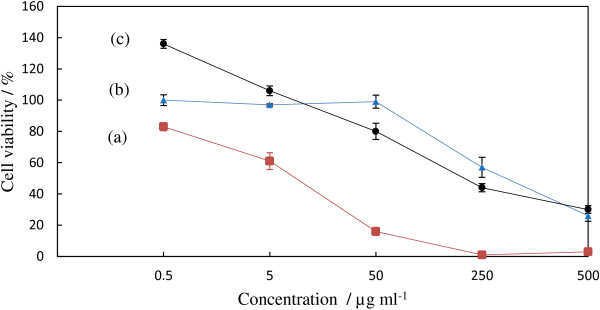
**Dose response curve of A549 cell proliferation throughout 72 hours. (a)** intercalation compound, Z-CFX. **(b)** free drug molecule, CFX. **(c)** host material, LZH.

Figure 10 shows the A549 cancer cell proliferation profiles after treatment with the intercalation compound, Z–CFX, the host, LZH and free drug, CFX for 72 h. Cell viability was measured using the MTT assay, which is based on the reduction of yellow tetrazolium MTT salt by metabolically active cells, leading to the formation of purple formazan crystals [54]. In general, all the three samples exerted toxic effects towards the A549 cells as the concentration increases. Upon comparing the IC_50_; the concentration that inhibits 50% of the cellular growth, Z-CFX shows lower IC_50_ compared with the value of free drug; 18.2 ± 3.2 μg/mL and 78.3 ± 2.5 μg/mL, respectively. This finding indicates that the compound has higher inhibitory effects towards the A549 cells compared to that of free drug. It is worth mentioning that precursor LZH has significantly as much as 8 times less toxicity than the intercalation compound (Figure 9c).

Al Ali et al. [47] found that zinc layered hydroxides intercalated with hippuric acid possessed synergistic effects with tamoxifen towards HepG2 cells in which the IC_50_ value significantly decreased than that of tamoxifen and hippuric acid alone. Li et al. [33] pointed out that decreased viability of HeLa cancer cells was due to LDH intercalation with folic acid which protected the anticancer drug from degradation and enhanced its permeability into the target cells. Considering the sustained release behavior of Z–CFX, we would attribute the enhanced antiproliferative effects observed in Z-CFX is due to the strong interactions occurred between LZH and CFX; the host would facilitate the cell uptake and further protect the guest from degradation so that the anions were slowly released and “killed” the A549 cells [55].

## Conclusions

CFX was successfully intercalated into the interlayers of layered zinc hydroxides via anion exchange mechanism in water:organic solvent mixture solution. The basal spacing of LZH was expanded to maximize the drug–host interactions in the intercalation compound, Z–CFX in which the intercalated CFX anions were bonded to tetrahedral Zn^2+^ moieties of the lattice in a unidentate coordination mode. Due to strong coordination bond between drug–host lattice, the intercalated anions were slowly released, following diffusion–anion exchange mechanisms in which diffusion from the interlayer anions being the rate limiting step. The antiproliferative towards A549 cells were enhanced due to the synergistic effects between CFX and LZH. These findings should serve as strong foundations in further development of biocompatible LZH-based drug carrier.

## Abbreviations

LHS: Layered hydroxide salts; LZH: Layered zinc hydroxide nitrate; CFX: Ciprofloxacin; Z–CFX: Layered zinc hydroxide nitrate intercalated with ciprofloxacin; H: Hour/hours; °C: Celcius degree; g: Gram; XRD: Powder x-ray diffraction; L: Liter; mL: Milliliter; FTIR: Fourier transform infrared; TGA: Thermogravimetric analysis; PBS: Phosphate-buffered saline; ZnO: Zinc oxide; Min: Minutes; BET: Brunauer–Emmett–Teller; BJH: Barrett–Joyner–Helenda.

## Competing interests

The authors declare they have no competing interests.

## Authors’ contributions

AFL prepared materials, analyzed and interpreted data and worked on the manuscript. MZH proposed the research and convened scientific personnels and assisted in the manuscript write-ups. JS oversaw the progress of toxicity screenings while WCC conducted the MTT assay and contributed to data interpretation. RA helped in TGA and reviewed the manuscript. All authors read and approved the final manuscript.

## Authors’ information

Prof. Dr. Mohd Zobir Hussein is professor of chemistry at the Institute of Advanced Technology, Universiti Putra Malaysia. His major research areas include layered organic–inorganic nanohybrid for gene and drug delivery, nanoparticles and nanostructured materials, their design, synthesis and applications. He has contributed to more than 200 technical papers. He is the assignor of 1 granted patent on the preparation method of nanomaterial for controlled release formulation and co-assignor of another 2 granted patents. In December 2012, he was awarded as one of Malaysia’s top scientist researcher by the government of Malaysia.
